# Supplementary material for: Lactic acid containing polymers produced in engineered Sinorhizobium meliloti and Pseudomonas putida
Source: PLoS One. 2020 Mar 19;15(3):e0218302. doi: 10.1371/journal.pone.0218302 (PMC7082056; doi:10.1371/journal.pone.0218302)
Supplement: Attachment — Submitted filename: Response to reviewers Tam PLOS rev.pdf [file pone.0218302.s002.pdf]

Second Revisions for Lactic Acid Containing Polymers Produced in Engineered *Sinorhizobium meliloti* and *Pseudomonas putida*

We have addressed Reviewer 3's concerns as follows.

Some issues should be corrected before acceptance:

1) Material and methods need to be adapted to the shortens version of this article, e.g. no data is shown for P. putida grown in liquid LB medium for polymer production, however this is still part of this section

   We have removed the information as appropriate.

2) The number of analyzed replicates should be stated, either in the figure or table legends or in the Material and methods section

   This information has been added where it had been missing.

3) p. 11 l. 195/196: how can there be error bars in a table? Delete this sentence

   This has been corrected.

4) p.15 l. 238 and table 3: see above and numbers are not presented with +/- standard deviation. Why do the authors use a different nomenclature for the P. putida strains in this table? Or are these other strains

   We have added standard deviation values and changed the strain nomenclature to be more consistent.

5) p. 17 ll. 314-320: This sentence is hard to understand as it is very long.

   We have revised the sentence structure.
