# Supplementary material for: Lactic acid containing polymers produced in engineered Sinorhizobium meliloti and Pseudomonas putida
Source: PLoS One. 2020 Mar 19;15(3):e0218302. doi: 10.1371/journal.pone.0218302 (PMC7082056; doi:10.1371/journal.pone.0218302)
Supplement: Attachment — Submitted filename: Response to reviewers Tam PLOS.docx [file pone.0218302.s001.docx]

**Revisions for Lactic Acid Containing Polymers Produced in Engineered *Sinorhizobium meliloti* and *Pseudomonas putida***

**Comments by Reviewer #1:**

The manuscript describes the production of lactic acid-containing polymers with medium-chain length units in Sinorhizobium meliloti and Pseudomonas putida. Many cases have been reported in various monomeric constituents using some bacterial platforms starting from 2008. In such a case, this is an example of the series study. Most recently Goto et al. reported the similar paper on the polymer with higher molar-fraction of lactic acid (> 4% in this paper) using Escherichia coli. Thy also provides the polymer properties. On the other hands, any new finding cannot be found in this paper. For example, in several points, new synthetic pathway, higher production titter, enriched lactic fraction, high molecular weight, new polymer properties. The reviewer has to consider this as a not-permitted assessment for publication in the present form.

**Response:**
We thank the reviewer for this comment. As stated in the comment, our study is another instance of a continuing effort to seek out new platforms for novel polymer production. There have been many reports using *Escherichia coli* as a host to produce different types of biopolymer (a given example is the paper of Goto et al. mentioned by the reviewer, and which we have now cited). However, as mentioned in our manuscript, using *E. coli* as a host has shown some drawbacks, such as lacking native system for polymer precursor production or suffering from the stress of polymer production. Therefore, our study has demonstrated the feasibility to produce these types of polymers in native polymer producers such as *Sinorhizobium meliloti* and *Pseudomonas putida.*

**Comments by Reviewer #2:**

General Comments

This manuscript describes the engineering of Sinorhizobium meliloti and Pseudomonas putida to synthesize novel co-polymers containing both polyhydroxyalkanoates (PHAs) and polylactic acid (PLAs). This work is novel and innovative. The manuscript is well written, the experimental approach and methods are appropriate and well described, and the conclusions are supported by the data. This manuscript will make an excellent contribution to the literature. There are only a few minor issues that need to be addressed before the manuscript may be published.

**General response**:

We apologize for those minor issues. We have addressed them as described following each comment.

Specific Comments

Page 6, line 102: In the sentence, “…were provided by R. Nordeste, who constructed them as recently described (27)…”, please provide the institutional affiliation of R. Nordeste.

**Response:**

R. Nordeste, University of Waterloo. This has been added to the manuscript.

Page 7, line 115: Please provide the vendor/manufacturer and its location for the culture medium “0.5*E2”

**Response:**

We have added the reference for 0.5X E2.

Page 7, lines 120 to 121: In the sentence, “β-glucuronidase levels were determined as an indication of the transcript level of synthesized genes in both E. coli and S. meliloti strains.” Firstly, do the authors really mean E. coli here? Should this not be P. putida?

Second, the assay described is protein (gene product) based. The authors cite two references, one which describes the use of the β-glucuronidase (GusA) assay in actinomycetes (reference 36) and the other describes the use of this assay as a measure of promoter activity in Lactic Acid bacteria (reference 37). In the current paper, the authors suggest that the assay is a direct indicator of the level of transcription of the genes of interest, but this is based on the assumption that the level of protein activity detected is proportional to the level of translation, and this in turn is proportional to the level of transcription. Is this actually the case? Can the authors provide some additional clarification about the relationship between transcription, translation, and enzyme activity in S. meliloti and P. putida?

**Response:**

We should be more clear about the rationale for this experiment. At first, we aimed to develop the plasmid-based system in our primary short-chain-length PHA producer host *S. meliloti.* Therefore, we conducted this experiment in *S. meliloti* only. We added *E. coli* as a positive control along with our host because the reporter gene was originally obtained from *E. coli.*

Regarding the second question, we again performed the assay in *E. coli* and *S. meliloti* to determine the transcriptional level of the genes of interest. The assay has been applied to quantitatively analyse the activity of the promoter (in terms of *gusA* expression), hence inferring the transcription of the genes that are constructed under the same promoter with the *gusA* gene fusions. This assay has been used in different systems such as plants, mosses, algae, fungi and various bacteria (*E. coli*, lactic acid bacteria, actinomycetes). We obtained similar results in *S. meliloti* compared to *E. coli* (data not shown) which strongly suggested the validity of this assay in our host.

Figure 3: The resolution of the image for Figure 3 is very poor, and the bands in each lane have merge with each other so it is difficult to tell which lane is which. Can the authors replace this gel image with a better one?

**Response:**

We have removed this figure, as we agree that the quality is poor, and it does not provide much useful information.

Page 11, lines 202 to 209: The authors refer to GusA, which is the gene product β-glucuronidase. For clarity, please revised the sentence on page 7, line 120, as follows: “β-glucuronidase (GusA) levels were determined….

**Response:**

We have edited the sentence at the reviewer’s suggestion.

**Comments by Reviewer #3:**

The authors present a plasmid containing the genes encoding the propionate CoA transferase of C. propionicum and a modified PHA synthase I of Pseudomonas sp. MBEL 6-19. They introduced this into phaC mutants of S. melioti and P. putida and report the production of PLA homopolymer in S. meloti and of a novel quaterpolymer P(3HB-co-LA-co-3HH-co-3HO) in P. putida in the presence of octanoate. Though the production of a novel quaterpolymer is potentially interesting for applied processes, the manuscript, in its present state, has a number of shortcomings.

**General response**:

We apologize for these shortcomings. We have addressed them as described following each comment.

p. 9, ll. 147-152: It would be helpful to have some more information on the genetic arrangement of the plasmid, e.g. how does the gusA reporter work, is it a transcriptional or translational fusion? How was the codon optimization achieved? What does the 532 or the 1400 stand for in the gene names? What RBSs are used and where were they located?

**Response:**

Figure 1 describes the genetic arrangement of the plasmid. It is a transcriptional fusion driven by the *tac* promoter under the regulation of *lacI^q^* on the vector; both genes of interest were introduced into the multiple cloning site (MCS) in front of the *gusA* gene. We have described how we constructed the plasmid in the text.

We described previously (Tran and Charles 2015) that these genes were codon-optimized for *S. meliloti* using the codon adaptation tool JCat (Grote et al., 2005). The optimized sequences, with a His tag of six histidine residues at the end of each gene (before the stop codon), and a *S. meliloti* ribosome binding site (RBS) at the beginning of each gene, were then synthesized on a single fragment, with *phaC1400* immediately following *pct532*. The *pct* gene which was originally from *Clostridium propionicum* had an increase of GC content from 40.38% to 53.65% after codon-optimization. Meanwhile, the *phaC* gene which was originally from *P. putida* did not show any drastic change of GC content (62.14% compared to initial 58.86%) after codon-optimization since *P. putida* has GC content similar to *S. meliloti*. In contrast, the overall number of codons drastically decreased across both genes (for the *pct* gene, the number of codons decreased from 52 to 37; for the *phaC* gene, the number of codon decreased from 56 to 39). Both numbers (532 and 1400) are used to be consistent with the gene names used by Jung et al. who originally mutated these genes and then screened them for altered enzyme activities.

Grote A, Hiller K, Scheer M, Munch R, Nortemann B, Hempel DC, Jahn D. 2005. JCat : a novel tool to adapt codon usage of a target gene to its potential expression host. Nucleic Acids Res. 33:526–531.

Jung YK, Kim TY, Park SJ, Lee SY. Metabolic engineering of Escherichia coli for the production of polylactic acid and its copolymers. Biotechnol Bioeng [Internet]. 2010;105(1):161–71.

p. 9, ll. 161-167 and Fig. 2: Why is there such a big difference in the fluorescence of SmUW257 comparing Fig. 2a to b? It should be comparable, as only IPTG is added in a) but this strain carries the empty plasmid. At the same time in 2b) it is hard to see a difference between the negative control (SmUW255) and the positive control (SmUW257). In my opinion this assay is not suitable to draw quantitative conclusions, only qualitative if any, as fluorescence depends on the amount and density of cells, which is hard to equal in solid phase medium. To get a rough estimation on the amount of polymer produced, the authors could repeat this plate experiment ensuring that the same amount of cells is plated or stain liquid cultures with the same OD (better cell count).

**Response:**

We also agree with the reviewer that this assay should only be used to qualitatively determine polymer production in bacteria. We used this technique as our initial rapid screening for assessment of polymer production in the different strains. For quantitative analysis, we used Gas Chromatography method to determine how much polymer was produced, as well as to identify polymer composition.

p.10, ll. 174-179 and Fig. 3: In MHO nothing can be concluded form this WB. Apart from the fact, that it is hard to distinguish the different lanes, no band is observed in the positive control, and the rest is mainly smear. How did the authors ensure that the same amount of protein is loaded in each lane, this should be stated in the M&M part or a Coomassie stained gel should be shown. Which lanes show the sample without IPTG, which is mentioned in the manuscript?

**Response:**

We have removed the figure.

p. 10, Polymer production (Fig.4): How many independent replicates where analyzed? What do the error bars represent? Is the % polymer referred to CDW?

**Response:**

The experiment was done in biological duplicate. Error bars represent the range of the mean. The % polymer content is referred to DCW.

p. 11, Tab. 2: Why is the PLA production negatively correlated to the lactic acid concentration in the medium? Is that expected? Please comment on this.

**Response:**

Even though lactic acid is theoretically an ideal substrate to produce polymer precursor, it does not seem to be a good substrate to support cell growth. Also, the more lactic acid we added, the more adverse effects on growth were observed, perhaps due to lowering pH in the medium. Evidently, we observed a significant decrease in DCW, as shown in Table 2. As a consequence, the overall PLA production is inversely proportional to the lactic acid concentration.

Fig. 5 and 6: Information on how many replicates were analyzed and what the error bar represent is missing. It would be more coherent to have in Fig. 5b and 6b the same order of the samples (from day 1 to 3 or vice versa).

**Response:**

The figures have been revised, as recommended. The experiment was done in duplicate. Results are presented as mean ± standard deviation (SD). Error bars indicate SD.

p. 12, ll. 227-228: The authors state that the gusA reporter expression is inversely proportional to PHB production. I don’t see a correlation between the expression and the polymer content. The GusA activity, which serves a s a proxy for gene expression, seems to be either on or off. The authors should perform a correlation and include a correlation coefficient to support this statement

**Response:**

We thank the reviewer for this comment. What we observed in our experiment that the longer we induced the promoter by adding IPTG at different time points, the lower polymer content detected there appeared to be. Meanwhile GusA activity increased in terms of induction time. More importantly, the strains were still able to produce polymer quite efficiently at base level (without induction). It is merely a suggestion that enzyme activity might not always be directly proportional to polymer production.

p.12, l. 229: Reference is made to S1, however it is not available.

**Response:**

We have removed this reference to S1

p.12, l. 230: How was the significance determined?

**Response:**

We have altered the statement to better reflect that we did not determine statistical significance.

Fig. 7a is redundant with Fig. 7b. and can be omitted from the manuscript.

It has been omitted.

p.14, l. 265: Did the authors mean sodium octanoate?

**Response:**

It is indeed sodium octanoate.

Fig 9a: When were the samples taken? Is this result of only one analysis? Please comment on this.

**Response:**

The time of sampling is provided in the Methods section. The experiment was done in duplicate.

Fig. 9b: How do the authors explain the difference in CDW in PPU20, PPU21, and PPU19? PPU19 reaches more than twice the CDW of the wt strain in LB medium. Why is this not observed with minimal medium? Is that a common phenotype, or just observed in this specific experiment?

**Response:**

We have removed these data from the paper.

Technical comments:
1. Information on media composition is completely missing (YM, TY, 0.5*E2)

**Response:**

We have provided references.

2. A list of abbreviations would be helpful (especially for the polymer abbreviations, HB, LA, HH. HV etc.).

**Response:**

We have now provided this on first use within the text.
